# No evidence for association of inherited variation in genes involved in mitosis and percent mammographic density

**DOI:** 10.1186/bcr3088

**Published:** 2012-01-07

**Authors:** Celine M Vachon, Jingmei Li, Christopher G Scott, Per Hall, Kamila Czene, Xianshu Wang, Jianjun Liu, Zachary S Fredericksen, David N Rider, Fang-Fang Wu, Janet E Olson, Julie M Cunningham, Kristen N Stevens, Thomas A Sellers, Shane V Pankratz, Fergus J Couch

**Affiliations:** 1 Department of Health Sciences Research, Mayo Clinic College of Medicine, Rochester, MN 55905, USA; 2 Department of Laboratory Medicine and Pathology, Mayo Clinic College of Medicine, Rochester, MN 55905, USA; 3 Department of Medical Epidemiology and Biostatistics, Karolinska Institute, Box 281, Stockholm 17177, Sweden; 4 Human Genetics, Genome Institute of Singapore, Singapore 138672; 5 Department of Epidemiology and Genetics, Moffitt Cancer Center, Tampa, FL 33612, USA

## Abstract

**Introduction:**

Increased mammographic breast density is one of the strongest risk factors for breast cancer. While two-thirds of the variation in mammographic density appears to be genetically influenced, few variants have been identified. We examined the association of inherited variation in genes from pathways that mediate cell division with percent mammographic density (PMD) adjusted for age, body mass index (BMI) and postmenopausal hormones, in two studies of healthy postmenopausal women.

**Methods:**

We investigated 2,058 single nucleotide polymorphisms (SNPs) in 378 genes involved in regulation of mitosis for associations with adjusted PMD among 484 unaffected postmenopausal controls (without breast cancer) from the Mayo Clinic Breast Cancer Study (MCBCS) and replicated the findings in postmenopausal controls (n = 726) from the Singapore and Sweden Breast Cancer Study (SASBAC) study. PMD was assessed in both studies by a computer-thresholding method (Cumulus) and linear regression approaches were used to assess the association of SNPs and PMD, adjusted for age, BMI and postmenopausal hormones. A *P*-value threshold of 4.2 × 10^-5 ^based on a Bonferroni correction of effective number of independent tests was used for statistical significance. Further, a pathway-level analysis was conducted of all 378 genes using the self-contained gene-set analysis method GLOSSI.

**Results:**

A variant in *PRPF4*, rs10733604, was significantly associated with adjusted PMD in the MCBCS (*P *= 2.7 × 10^-7^), otherwise, no single SNP was associated with PMD. Additionally, the pathway analysis provided no evidence of enrichment in the number of associations observed between SNPs in the mitotic genes and PMD (*P *= 0.60). We evaluated rs10733604 (*PRPF4*), and 73 other SNPs at *P *< 0.05 from 51 genes in the SASBAC study. There was no evidence of an association of rs10733604 (*PRPF4*) with adjusted PMD in SASBAC (*P *= 0.23). There were, however, consistent associations (*P *< 0.05) of variants at the putative locus, *LOC375190*, Aurora B kinase (*AURKB*), and Mini-chromosome maintenance complex component 3 (*MCM3*) with adjusted PMD, although these were not statistically significant.

**Conclusions:**

Our findings do not support a role of inherited variation in genes involved in regulation of cell division and adjusted percent mammographic density in postmenopausal women.

## Introduction

Mammographic density is a trait that represents the proportion of stromal and epithelial tissues in a radiographic image of the breast. Women with **>**50% dense tissue are at an estimated four- to six-fold increased risk of breast cancer relative to those with **<**10% [1,2].

Little is known about the biology of mammographic density or the mechanisms underlying the association between density and breast cancer. However, there is mounting evidence that genetic influences account for a large proportion of variation in mammographic density [3-5]. Indeed, it has been estimated that 61% to 67% of the variation in percent mammographic density adjusted for age and covariates, may be attributable to genetics [3]. To date, few loci have been shown associated with the mammographic density measures that predict breast cancer [6]. A recent study by Odefrey and colleagues [7] confirmed the association of a variant (rs3817198) in lymphocyte-specific protein 1, *LSP1*, with mammographic density. Importantly, this variant was initially identified as a breast cancer susceptibility locus [8]. Also, the first meta-analysis of genome-wide association studies of adjusted percent mammographic density identified an association with rs10995190 in *ZNF365 *[9], which has also been shown to be a risk factor for breast cancer [10]. Although these loci are promising, they explain little variation in the density measures and suggest other genetic variation for mammographic density remains to be determined.

Mammographic density has been hypothesized to reflect the cumulative exposure of breast stroma and epithelium to hormones and growth factors that can stimulate cell division and proliferation [11]. Evidence for this can be seen in the multiple studies showing positive associations of mammographic density with use of postmenopausal hormone therapy (PMH), especially estrogen and progesterone therapy [12-19], as well as with circulating IGF-1 (in premenopausal women) [20-23], both of which have been shown to exert proliferative effects on the breast. In one study of PMH, density, and tissue characteristics, PMH was associated with increased density, greater fibrous stroma, and less complex lobule type (lobule type 1), independent of estrogen and progesterone receptor up-regulation [24]. Increased density was also associated with Ki67 activity in the ducts and lobules [24], although this has not been confirmed in the majority of other studies [25-28]. These findings are consistent with the evidence that fibrous stroma differentiates dense and non-dense breast tissue [28-32]. Recent histologic studies that have compared targeted regions of dense and non-dense tissue in healthy patients suggest the proportion of connective tissue and relative cellularity of stromal cells is higher in dense vs. non-dense areas of the breast; this was not consistently seen or was seen to a lesser extent for the epithelial tissues [28,33]. We have also shown increased aromatase expression in stromal cells from dense vs. non-dense areas of the breast, which could result in increased production of estrogens, and consequent stimulation of cellular proliferation [34]. Furthermore, stromal cells can produce growth factors such as IGF-1 that may also stimulate proliferation through paracrine mechanisms [24,35].

Genes involved in regulation of cell division or mitosis could mediate the influence of these endogenous and exogenous exposures on breast tissue, reflected in variations in mammographic density. For instance, virgin Sprague-Dawley rats treated with the placental hormone human chorionic gonadotropin (hCG) to mimic pregnancy show unique genomic signatures, including expression of genes involved in cell division control, which were not seen in rats receiving 17beta-estradiol and progesterone [36]. In addition, non-epithelial nuclear area, which may represent increased nuclear size due to failed or delayed cell division, has been associated with mammographic breast density in women over age 50 [37]. Here we present a comprehensive analysis of the association between variation in 378 genes involved in regulation of mitosis and mammographic density in postmenopausal women.

## Materials and methods

### Study population

The Mayo Clinic Breast Cancer Study (MCBCS) is an on-going clinic-based case-control study initiated in February 2001 at Mayo Clinic, Rochester, MN. Details of the study design and data collection procedures have been previously described [38]. Briefly, cases were women over age 20 years with histologically confirmed primary invasive breast carcinoma enrolled within six months of the date of diagnosis. Controls without prior history of cancer (other than non-melanoma skin cancer) were matched on age (± 5 years) and region of residence to cases. Controls were selected from the outpatient clinic in the Department of Internal Medicine at Mayo Clinic where they were seen for general medical examinations. A self-administered risk factor questionnaire, blood sample, permission to obtain mammograms and written informed consent were obtained from all participants. Case participation was 69% (n = 798 cases) and control participation was 71% (n = 843 controls). All subjects provided written, informed consent and the protocol was reviewed by the Mayo Clinic Institutional Review Board.

### Mammographic density measurement

Mammograms were only ascertained on controls for this analysis, as the focus of the study was to understand the genetics of mammographic density among healthy women. The closest screening mammogram to enrollment date (median 0 days, 59% were same day, 82% within 1 year) was obtained and digitized on a Kodak Lumiscan 75 scanner (LS 75) (Lumisys/Eastman Kodak Co, Rochester, New York, USA) with 12-bit grayscale pixel depth for 686 of the 843 (81%) control women; analyses focused on the 484 mammograms (of 579 postmen eligible or 84%) from postmenopausal Caucasian women due to the composition of the SASBAC replication study. We estimated mammographic density using the cranial-caudal (CC) or top-down view from the left breast using a validated computer-assisted thresholding program (Cumulus [39] University of Toronto, Toronto, Ontario, Canada) that we have used in previous reports [40-42] and has been shown by our group to predict breast cancer [43]. We assessed percent mammographic density defined as the absolute area of dense tissue on the mammogram divided by the total area multiplied by 100. All images were read by one trained technician who consistently maintained high reliability (r > 0.90) while reading duplicate images across varying time frames [41,43].

### Gene and SNP selection

We identified genes encoding proteins involved in regulation of all aspects of cell division, identified through the literature and known pathways. Specifically, we chose genes implicated in mitotic entry, mitotic progression, the mitotic checkpoint, cytokinesis and mitotic exit. In addition, we included genes implicated in mitotic function through functional screens [44,45] and genes involved in the structure and function of centrosomes [46], which are directly involved in chromosome segregation. SNPs representing common genetic variation within these 378 genes were identified and examined with percent mammographic density. SNP selection has been described in detail elsewhere [38,46]. Briefly, we first selected tagSNPs (r^2 ^> 0.80) from SNPs with MAF ≥ 0.05 located within 5 kb of the largest cDNA isoform (genome build 35) to represent a reduced set of SNPs in each gene [47]. We prioritized putative functional SNPs (within 1 kb upstream, 5' UTR, 3' UTR or non-synonymous) with MAF ≥ 0.05 identified in Ensembl version 34. A total of 2,058 SNPs in 378 genes were identified.

As detailed elsewhere [38], samples from both cases and controls, (including 5% duplicate samples), were assayed at Illumina Corporation (San Diego, CA, USA) on an Illumina BeadLab using the Illumina GoldenGate Assay™. DNA activation, incubation with assay oligonucleotides, PCR amplification and analysis using the BeadStudio software for automated genotype clustering and calling was performed according to a standard protocol [48-50]. Successful genotyping was achieved for 99.9% of DNA samples (seven case DNAs failed). Analyses of SNPs with mammographic density were limited to postmenopausal controls. We assessed departures from Hardy-Weinberg equilibrium (HWE) (*P *< 0.001) in these 484 postmenopausal control subjects using a Pearson goodness-of-fit test. Of the 2,058 SNPs genotyped, 2,048 (99.5%) were in HWE, and SNP call rates were > 99% in 2,041 SNPs (2,053 SNPs > 98%). Also, only 24 (5%) of the 484 had sample call rates below 98%, but these were all above 95%.

### SASBAC study

The Singapore and Sweden Breast Cancer Study (SASBAC) is a population-based case-control study of postmenopausal breast cancer in women aged 50 to 74 years born in Sweden. Details on data collection and subjects have been described previously [51]. Controls were white Europeans randomly selected from the Swedish population and frequency matched to the expected age distribution of cases and on geographical area. They served as the replication sample for this study. The final study group with both mammographic density and genotype data included 726 controls of 764 eligible (95%). Approval of the study was given by the ethical review board at the Karolinska Institute (Stockholm, Sweden) and six other ethical review boards in the respective regions in which the subjects were based.

Screening film mammograms corresponding to the enrollment date were obtained. The medio-lateral oblique (MLO) view was digitized using an Array 2905HD Laser Film Digitizer (Array Corporation, Roden, The Netherlands, which covers a range of 0 to 4.7 optical density. Similar to the MCBCS, the Cumulus software was used for determination of percent mammographic density on a randomly selected side. A random 10% of the images were included as replicates to assess the intra-observer reliability, which was high with a Spearman rank correlation coefficient of 0.95.

Associations of any SNP with *P *< 0.05 in MCBCS by the log-additive test were attempted for *in silico *replication within SASBAC using available genotype information from a genome wide association study (GWAS) of breast cancer [52]. Briefly, 764 controls were genotyped on the HumanHap550 BeadChip; of these, 726 (95%) controls had films available. When the exact SNP was genotyped and available as part of the SASBAC GWAS (which occurred 56% of the time), the corresponding *P*-value for that SNP was used. If the exact SNP was not available, we examined the association with available SNPs in high linkage disequilibrium (LD), defined as r^2 ^> 0.70 with the SNP of interest, using HapMap CEU.

### Mammographic density comparison studies

**Figure 1:**
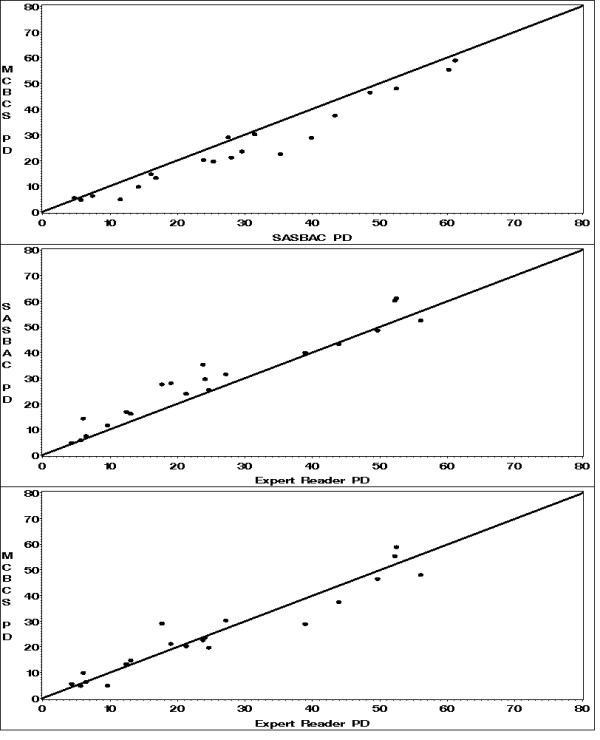
**Comparison of percent mammographic density estimation among MCBCS, SASBAC, and expert readers**.

We compared the similarity of percent mammographic density (PMD) assessment between the readers from MCBCS and SASBAC using a standard set of 20 film mammogram images across varying densities. The intra-reader reliability assessed as the intraclass correlation or ICC for PMD between readers was high (ICC = 0.99; Figure 1). We also assessed the intraclass correlations between our readers with Dr. Norman Boyd, an expert in the estimation of density and found strong agreement (ICC = 0.98 and 0.99 for MCBCS and SASBAC, respectively, Figure 1).

**Figure 2:**
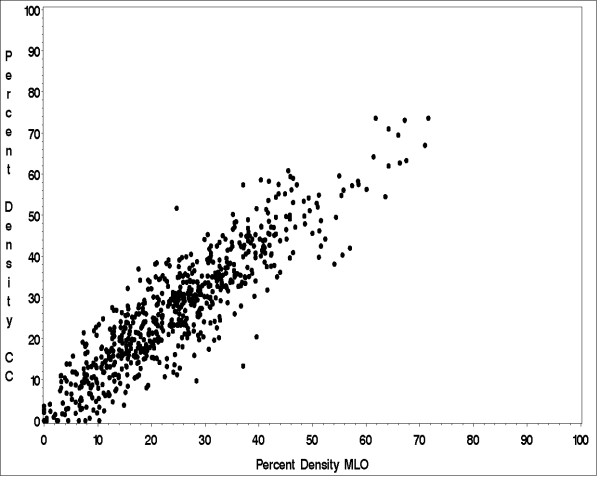
**Comparison of percent mammographic density estimation on MLO vs. CC views from 700 controls **[43].

Further, since MCBCS and SASBAC ascertained and estimated PMD from different mammogram views (CC vs. MLO, respectively), we were interested in the differences in PMD between the two mammogram views. A previous study of 30 women found strong correlations of CC and MLO views (between 0.86 and 0.96), suggesting representative information is provided in a single view [53]. We conducted a larger study of 700 controls with both right and left CC and MLO views [43]. We examined the differences in mean PMD as well as Pearson correlations (r) assessed from the CC and MLO views from the same breast. We found the average absolute difference in PMD between CC and MLO views to be 2.0% (SD = 6.5) for the left and 2.2% (SD = 6.4%) for the right breast. The Pearson correlations between the PMD from CC and MLO were also very high, with r = 0.90 for both left and right breasts (Figure 2).

### Statistical analysis

Primary analyses focused on the 484 postmenopausal controls in the MCBCS study, since SASBAC was comprised only of postmenopausal women. Initially, we examined the distribution of risk factors and mammographic density among postmenopausal controls from MCBCS and SASBAC. Genotypes from controls were used to estimate allele frequencies within each study set.

Individual SNP associations with PMD were assessed using linear regression. No transformation of PMD was made in MCBCS since residuals were approximately normal. Tests for associations were carried out assuming an ordinal (log-additive or additive) genotypic relationship using simple tests for trend within the linear regression models. All analyses were adjusted for age, body mass index (BMI) and current post-menopausal hormone (PMH) use. Examination of transformations of BMI and age did not result in substantial improvement in model fit when compared to models that were based on the original scale of these covariates. Because of this, and because linear regression assumptions were met in the analysis models, the original scale for these variables was used in all analyses on MCBCS data.

Similar analyses were performed for the replication of SNPs in the SASBAC sample with PMD, although a square root transformation of PMD was required to meet linear regression assumptions. Analyses were adjusted as above. Mean PMD from SASBAC was back-transformed for each genotype within the context of the general model in order to more directly compare results to those from MCBCS.

A pathway-level analysis was conducted using the self-contained gene-set analysis method, GLOSSI [54]. This algorithm, based on Fisher's combined probability test, is designed to determine if the distribution of *P*-values in a set of genes deviates from what is expected based on the null hypothesis of no association. GLOSSI was implemented using 2,028 SNPs from the 378 genes among the 484 postmenopausal breast cancer-free controls in the MCBCS study to test for an association with this pathway and PMD adjusted for age, BMI and PMH use as above. A pathway-level *P*-value was obtained based on 500 permutations.

To assess heterogeneity of associations by study, a meta-analysis was performed on the ordinal parameter estimates and the Q-test was calculated [55]. In order to make the ordinal estimates comparable between studies for the meta-analysis, ordinal estimates for the MCBCS sample were estimated using square root transformed percent density within this sample. These parameter estimates were used to calculate the Q-test statistic and resulting heterogeneity *P*-value.

In order to set a threshold for statistical significance that appropriately reflects the number of SNPs tested, recognizing that SNPs within genes may not be independent, we calculated an effective number of independent tests within each gene using an eigenvalue based measure as proposed by Galwey [56]. We summed the effective number of independent tests per gene across all genes in the study to estimate the effective number of independent tests (n = 1,178 for stage I and n = 64 for replication). We use this result to set our threshold for significance via a Bonferroni correction for the number of independent tests (0.05/1,178 = 4.2 × 10^-5 ^for stage I and 0.05/64 = 7.8 × 10^-4 ^for replication). Analyses were implemented using SAS (SAS Institute, Cary, NC, USA, Version 8, 1999), S-Plus (Insightful Corp, Seattle, WA, USA, Version 7.05, 2005) and R software systems.

## Results

The 2,058 SNPs from 378 genes were examined for associations with PMD among 484 postmenopausal women within MCBCS. Characteristics of the MCBCS postmenopausal controls are described in Table 1. One variant, rs10733604 in *PRPF4*, was associated with adjusted PMD (*P *= 2.7 × 10^-7^). A second, albeit not statistically significant, association was seen with rs12563929 in *PRKACB *(*P *= 2.2 × 10^-4^) (Additional file 1). In total, we found 88 SNPs in 58 genes associated at *P *< 0.05 with percent density. These SNPs were selected for examination within the SASBAC study (Additional file 1). The pathway analysis incorporating all 378 genes showed no evidence of enrichment in the number of associations observed between SNPs in the mitotic genes and PMD (*P *= 0.60).

**Table 1:** Characteristics of two postmenopausal control populations (MCBCS^a^, 2001-2005 and SASBAC^b^, 1993-1995).

Characteristic	Level	MCBCS^a^ (n = 484)		SASBAC^b^ (n = 726)	
		N	% or mean	N	% or mean
Age, years	40 to 49	26	5.4	1	0.1
	50 to 59	157	32.4	237	32.6
	60 to 69	176	36.4	363	50.0
	70+	125	25.8	125	17.2
Body mass index, kg/m^2^	Mean (SD)	464	26.9 (5.3)	718	25.7 (4.1)
Postmenopausal hormone use	Current	153	31.6	95	13.1
	Former	163	33.7	186	25.6
	Never	136	28.1	362	49.9
	Unknown	32	6.6	83	11.4
Percent Mammographic Density, (%)	Mean (SD)	484	18.6 (13.9)	726	14.4 (13.9)
Categories	0	30	6.2	45	6.2
	1 to 9	116	24.0	324	44.6
	10 to 24	201	41.5	225	31.0
	25 to 49	125	25.8	111	15.3
	50 to 74	11	2.3	21	2.9
	75+	1	0.2	0	0

^a^Mayo Clinic Breast Cancer Study. ^b^Singapore and Sweden Breast Cancer Study.

The 726 controls from SASBAC were slightly younger (mean age 62.8 ± 6.2 vs. 63.6 ± 9.2), less likely to use PMH (38.7% vs. 65.3%) and had lower average BMI (25.7 vs. 26.9) and PMD (14.4% vs. 18.6%) than the MCBCS controls (Table 1). Both studies showed inverse associations of age and BMI with PMD and positive associations with current PMH use (Table 2).

**Table 2:** Adjusted mean percent mammographic density by age, BMI, postmenopausal hormone use (MCBCS and SASBAC controls)^a^.

Characteristic	Level	MCBCS Controls (n = 484)		SASBAC Controls (n = 726)	
		
		Mean	95% CI	Mean	95%CI
Age, years	40 to 49	23.6	18.3 to 28.9	8.2	-------
	50 to 59	20.1	17.9 to 22.2	16.7	14.9 to 18.6
	60 to 69	18.2	16.1 to 20.2	13.7	12.4 to 15.1
	70+	16.4	14.0 to 18.8	12.0	9.5 to 14.5
Body mass index, kg/m^2b^	< 25	24.8	24.0 to 27.6	19.2	17.5 to 20.8
	25 to 30	16.0	14.2 to 17.9	11.2	10.0 to 12.5
	> 30	10.6	8.2 to 13.0	6.8	5.1 to 8.5
	Unknown	15.9	10.2 to 21.5	10.5	3.9 to 17.2
Postmenopausal hormone use^c^	Current	21.9	19.8 to 24.0	19.6	16.4 to 22.7
	Former	18.1	16.1 to 20.2	17.0	14.9 to 19.1
	Never	15.6	13.4 to 17.9	11.7	10.6 to 12.8
	Unknown	18.3	12.7 to 23.9	14.7	11.8 to 17.6

^a^Mayo Clinic Breast Cancer Study; Singapore and Sweden Breast Cancer Study.

^b ^Adjusted for age. ^c^Adjusted for age and BMI.

Within SASBAC, genotype information was available for 73 of the 88 SNPs (located in 51 genes) associated with PMD at *P *< 0.05 in MCBCS. Of these, 56% were the exact SNP but the remainder were SNPs in moderate to high linkage disequilibrium (LD > 0.70) with the SNP of interest within MCBCS. Both of the SNPs rs10733604 in *PRPF4 *and rs12563929 in *PRKACB *were available in SASBAC (noted in Additional file 1).

There were no associations of rs10733604 (*P *= 0.23) or rs12563929 (*P *= 0.93) with percent density in the SASBAC study. Eight of the 73 (10.9%) candidate SNPs, located in seven genes, displayed associations also at *P *< 0.05 with PMD in the SASBAC study (Table 3), although none reached the statistical threshold. Only variants in *LOC375190 *(rs2080727) *AURKB *(rs4792590 and rs3027260, LD of r^2 ^= 0.71) and *MCM3 *(rs3765447), showed consistent direction of effect in both the MCBCS and SASBAC studies, which was also reflected in the tests of heterogeneity (Table 3).

**Table 3:** Polymorphisms in mitotic pathway genes^a ^and percent mammographic density among MCBCS^b ^(n = 484) and SASBAC^b^(n = 726) controls.

					MCBCS			SASBAC			
						
Chr	Gene Name	Effect	SNP	Position (bp)	N	PMD Est (SE)/ adj mean^c^	*P*-value^d^	N	Sqrt PMD Est (SE)/ adj mean^e^	*P*-value^d^	P-Het^f^
1	*GALNT2*	Ordinal	rs1043908	228483917	484	2.88 (1.28)	0.025	725	-0.314 (0.135)	0.02	< 0.001
		A/A			373	17.9		555	19.0		
		A/G			102	20.8		158	16.3		
		G/G			9	23.6		12	13.9		
2	*LOC375190*	Ordinal	rs2080727	24204411	484	1.69 (0.85)	0.048	725	0.185 (0.095)	0.05	0.54
		A/A			195	17.2		305	17.1		
		A/G			215	19.4		332	18.7		
		G/G			74	20.2		88	20.3		
6	*MCM3*	Ordinal	rs3765447	52249471	483	3.62 (1.76)	0.040	725	0.45 (0.188)	0.017	0.96
		A/A			422	18.2		644	17.7		
		A/G			60	22.3		76	21.7		
		G/G			1	12.7		5	26.1		
8	*TNKS*	Ordinal	rs12549064	9479437	484	-2.15 (1.09)	0.048	725	0.321 (0.116)	0.006	0.001
		A/A			303	19.5		496	17.8		
		A/C			166	17.4		205	20.6		
		C/C			15	15.1		24	23.6		
10	*KIF11*	Ordinal	rs2275220	94362686	484	-4.23 (2.12)	0.046	726	0.45 (0.172)	0.009	0.005
		A/A			448	19.0		613	17.9		
		A/G			34	13.5		111	22.0		
		G/G			2	20.4		2	26.4		
11	*STIM1*	Ordinal	rs3794050	4068476	484	2.85 (1.38)	0.039	726	-0.383 (0.142)	0.007	0.003
		G/G			389	17.8		547	19.4		
		G/A			89	23.0		173	16.2		
		A/A			6	8.6		6	13.3		
17	*AURKB*	Ordinal	rs3027260	8053911	484	-3.33 (1.52)	0.029	726	-0.404 (0.177)	0.022	0.73
		G/G			404	19.2		632	18.7		
		G/A			77	15.8		91	15.4		
		A/A			3	12.8		3	12.4		
		Ordinal	rs4792590	8057840	484	-3.14 (1.34)	0.020	726	-0.39 (0.152)	0.010	0.89
		G/G			377	19.3		597	18.6		
		G/A			102	16.6		121	15.4		
		A/A			5	9.5		8	12.5		

^a^*P *≤ 0.05 in both studies. ^b^Mayo Clinic Breast Cancer Study; Singapore and Sweden Breast Cancer Study.

^c^PMD Est(SE): ordinal parameter estimate and standard error reflecting the estimated change in percent density per each additional copy of the minor allele carried. Adjusted mean from general model: least squares estimate of mean percent density for each genotype, adjusted for age, BMI, and PMH. ^d^*P*-value from analyses adjusted for age, BMI, and PMH. ^e^Sqrt PMD Est(SE): ordinal parameter estimate and standard error reflecting the estimated change in percent density error per each additional copy of the minor allele carried. Adjusted mean from general model: least squares estimate of mean percent density for each genotype, adjusted for age, BMI, and PMH. Least squares means back transformed from square root to original scale. ^f^*P*-value from test for heterogeneity from meta-analysis performed on the ordinal parameter estimates (ordinal estimates for the MCBCS sample were estimated using square root transformed PMD).

## Discussion

Overall, we found no statistically significant associations between SNPs involved in mitosis with percent mammographic density. There were consistent associations (albeit only at a significance of *P *< 0.05) among four SNPs in three genes involved in either cell division (*AURKB *AND *LOC375190*) or cellular proliferation (*MCM3*) and adjusted percent density among two studies of postmenopausal women. These associations warrant further investigation.

Strengths of this study include examination of a novel pathway with adjusted percent mammographic density, SNP associations examined in two independent populations, similar quantitative measures of density used in both studies, adjustment for potential confounding factors, and focus on the homogenous subgroup of postmenopausal, healthy women. Also, non-replication of associations in the SASBAC study did not appear to be due to systematic differences in the readers' performance or mammogram view assessed, as our comparison studies suggested estimates were similar for both readers across a common set of images as well as by MLO vs. CC views. However, limitations include inability to confirm associations for 15 SNPs and use of different digitizers that could potentially add systematic bias to the percent density estimation.

Although very little is known about the biology underlying mammographic density, evidence strongly suggests that genes contribute to a large proportion of the variation in density [3,4,7,9]. To date, replicated associations between genetic variants associated with a few candidate genes, including *IGF-1, ESR1, HSD3B1*, and *COMT *and breast density have been observed, implicating primarily pathways that regulate steroid hormone synthesis and metabolism, hormone receptors and proliferative pathways, including the insulin-like growth factor pathway [6]. Our study is the first to perform a comprehensive analysis of candidate genes in mitosis. However, our results do not suggest a strong role of genes involved in mitosis with percent mammographic density.

Interestingly, two of the genetic loci (*AURKB, LOC375190*) containing SNPs that displayed consistent, albeit nonsignificant, associations with adjusted percent mammographic density in the MCBCS and SASBAC studies, have been implicated in the regulation of the metaphase to anaphase transition during chromosome segregation. Although little is known about the protein encoded by *LOC375190*, a functional siRNA based screen has shown that reduced levels of *LOC375190 *causes severe spindle defects and mitotic arrest and subsequent formation of polyploid cells due to premature mitotic exit [44]. In contrast, much is known about the role of the *AURKB-*encoded Aurora B protein in mitotic regulation. While Aurora B has been implicated in mitotic entry and also in cytokinesis and mitotic exit, the primary role for this kinase is in the assembly of factors involved in spindle attachment and tension and regulation of the mitotic checkpoint. Loss or gain of Aurora B results in defects in the metaphase to anaphase transition and subsequent aneuploidy due to chromosome segregation defects or polyploidy due to premature mitotic exit. Interestingly, Aurora B is localized at centrosomes during mitosis and may influence spindle growth from the centrosome to the kinetochore during mitosis, similar to *LOC375190*, resulting in defects in chromosome segregation and/or premature mitotic exit [57]. Given these common functions, it is tempting to speculate that common genetic variation in these two loci may result in defects in chromosome segregation, premature mitotic exit and an increase in the number of cells with multiple nuclei.

Since non-epithelial nuclear area, which may represent increased nuclear size due to failed or delayed cell division, has been associated with mammographic breast density in women over age 50 [37], studies of cell division in dense and non-dense mammary tissues may provide further insight into the associations reported here. However, since these proteins are multifunctional and may influence other cellular processes, including growth factors [58], alternative explanations for the associations with density must also be considered.

The third locus of interest, a variant in mini-chromosome maintenance complex component 3 or *MCM3*, however, plays a role in cellular proliferation. The protein encoded by *MCM3 *is one of the highly conserved mini-chromosome maintenance proteins (MCM) that are involved in the initiation of genome replication. The acetylation of this protein inhibits the initiation of DNA replication and cell cycle progression. Proliferation of stroma and/or epithelium has been hypothesized to underlie increased mammographic density [11], although few studies have shown positive associations between proliferation markers and PMD [24,26,27,59] or tissue from dense areas of the breast [28].

## Conclusion

In summary, we present the first report of variation in genes involved in regulation of cell division and mammographic density. We find no strong evidence of association between variants in genes involved in mitosis and adjusted percent mammographic density; however, further investigation of variants in *AURKB, LOC375190 *and *MCM3 *is warranted.

## Abbreviations

*AURKB*: Aurora B Kinase; BMI: Body Mass Index; CC: cranio-caudal; CEU: One of 11 populations in the Hapmap (Northern and Western European Ancestry); GWAS: genome wide association study; hCG: human chorionic gonadotropin; HWE: Hardy-Weinberg equilibrium; LD: linkage disequilibrium; MCBCS: Mayo Clinic Breast Cancer Study; MCM3: mini-chromosome maintenance complex component 3; MLO: medio-lateral oblique; PMH: postmenopausal hormones; PMD: Percent Mammographic Density; SASBAC: Sweden and Singapore Breast Cancer Study; SNP: single nucleotide polymorphism

## Competing interests

The authors declare that they have no competing interests.

## Authors' contributions

CMV, FJC, JEO and TAS contributed to all aspects of the study, including study design, data collection, analyses, interpretation and preparation of the manuscript. XW, JMC and DNR were responsible for samples and genotyping for MCBCS. JLi, PH, KC and JLiu contributed to the SASBAC replication results, interpretation and publication. VSP, ZSF, CGS and JLi conducted statistical analyses and provided results. KNS performed the pathway analyses and provided interpretation of results and scientific review of the manuscript. FFW was responsible for all aspects of mammogram retrieval and mammographic density estimation. All authors read and approved the final manuscript.

## Supplementary Material

Additional file 1 **Associations between variants in genes in the mitotic pathway and percent mammographic density (PMD) in two studies of postmenopausal women.**484 Caucasian subjects from the Mayo Clinic Breast Cancer Study (MCBCS, 2001 to 2005) and 726 Caucasian subjects (controls) from Singapore and Sweden Breast Cancer Study (SASBC, 1993-1995). Click here for file
