# Supplementary material for: No evidence for association of inherited variation in genes involved in mitosis and percent mammographic density
Source: Breast Cancer Res. 2012 Jan 7;14(1):R7. doi: 10.1186/bcr3088 (PMC3496122; doi:10.1186/bcr3088)
Supplement: Additional file 1 — Associations between variants in genes in the mitotic pathway and percent mammographic density (PMD) in two studies of postmenopausal women.484 Caucasian subjects from the Mayo Clinic Breast Cancer Study (MCBCS, 2001 to 2005) and 726 Caucasian subjects (controls) from Singapore and Sweden Breast Cancer Study (SASBC, 1993-1995). [file bcr3088-S1.doc]

**Supplementary Table 1: Associations between variants **in genes in the mitotic pathway and percent mammographic density (PMD) in two studies of postmenopausal women. 484 Caucasian subjects from the Mayo Clinic Breast Cancer Study (MCBCS, 2001 to 2005) and 726 Caucasian subjects (controls) from Singapore and Sweden Breast Cancer Study (SASBC,** 1993-1995**)**.**

| **ChrGene Name** | **Effect** | **MCBCS** | **SASBAC^a^** | | | | |  | | | | | |
| --- | --- | --- | --- | --- | --- | --- | --- | --- | --- | --- | --- | --- | --- |
|  |  |  | **SNP** | **Position (bp)** | **N** | **PMD Est (SE)/**  **adj mean^b^** | P-value^c^ | **SNP** | **Position (bp)** | **LD** | **N** | **Sqrt PMD Est (SE)/**  **adj mean^d^** | P-value^c^ |
| 1 | *PRKACB* | Ordinal | **rs12563929** | **84320872** | **484** | **-5.93 (1.60)** | **0.0002** | **rs12563929** | **84320872** | **N/A** | **726** | **-0.017(0.188)** | **0.929** |
|  |  | A/A |  |  | **416** | **19.5** |  |  |  |  | **637** | **18.4** |  |
|  |  | A/G |  |  | **65** | **13.3** |  |  |  |  | **87** | **18.2** |  |
|  |  | G/G |  |  | **3** | **10.7** |  |  |  |  | **2** | **18.1** |  |
|  |  | Ordinal | rs12728744 | 84355143 | 483 | 4.04 (1.97) | 0.041 | rs12728744 | 84355143 | N/A | 726 | 0.075(0.183) | 0.682 |
|  |  | A/A |  |  | 433 | 18.2 |  |  |  |  | 642 | 18.3 |  |
|  |  | A/G |  |  | 50 | 22.3 |  |  |  |  | 81 | 18.9 |  |
|  |  | G/G |  |  | 0 |  |  |  |  |  | 3 | 19.6 |  |
|  |  | Ordinal | rs12129674 | 84385847 | 482 | -3.36 (1.24) | 0.007 |  |  |  |  |  |  |
|  |  | A/A |  |  | 368 | 19.4 |  |  |  |  |  |  |  |
|  |  | A/G |  |  | 104 | 17 |  |  |  |  |  |  |  |
|  |  | G/G |  |  | 10 | 8.6 |  |  |  |  |  |  |  |
|  |  | Ordinal | rs7547892 | 84431179 | 484 | 1.99 (0.83) | 0.017 | rs7547892 | 84431179 | N/A | 726 | 0.006(0.091) | 0.949 |
|  |  | A/A |  |  | 140 | 17.1 |  |  |  |  | 188 | 18.3 |  |
|  |  | A/G |  |  | 230 | 18.3 |  |  |  |  | 374 | 18.4 |  |
|  |  | G/G |  |  | 114 | 21.2 |  |  |  |  | 164 | 18.4 |  |
| 1 | *CDC14A* | Ordinal | rs524219 | 100594081 | 483 | -3.66 (1.89) | 0.053 | rs6577168 | 100578992 | 0.867 | 726 | 0.165(0.17) | 0.332 |
|  |  | C/C |  |  | 428 | 19 |  |  |  |  | 621 | 18.1 |  |
|  |  | C/G |  |  | 55 | 15.4 |  |  |  |  | 100 | 19.5 |  |
|  |  | G/G |  |  | 0 |  |  |  |  |  | 5 | 21 |  |
| 1 | *GPSM2* | Ordinal | rs12726126 | 109237651 | 484 | 2.70 (1.39) | 0.053 | rs17621851 | 109202358 | 1.0 | 725 | 0.166(0.145) | 0.253 |
|  |  | A/A |  |  | 397 | 18.2 |  |  |  |  | 579 | 18 |  |
|  |  | A/G |  |  | 80 | 20.2 |  |  |  |  | 136 | 19.4 |  |
|  |  | G/G |  |  | 7 | 26.6 |  |  |  |  | 10 | 20.9 |  |
| 1 | *NUF2* | Ordinal | rs1509021 | 161555168 | 484 | -1.72 (0.84) | 0.041 | rs10494387 | 161524157 | 0.967 | 726 | 0.089(0.09) | 0.324 |
|  |  | A/A |  |  | 123 | 19.6 |  |  |  |  | 178 | 17.6 |  |
|  |  | A/G |  |  | 238 | 19.4 |  |  |  |  | 363 | 18.4 |  |
|  |  | G/G |  |  | 123 | 16.1 |  |  |  |  | 185 | 19.1 |  |
|  |  | Ordinal | rs952089 | 161555290 | 484 | -2.67 (1.03) | 0.010 |  |  |  |  |  |  |
|  |  | A/A |  |  | 300 | 19.7 |  |  |  |  |  |  |  |
|  |  | A/G |  |  | 161 | 17.4 |  |  |  |  |  |  |  |
|  |  | G/G |  |  | 23 | 13.2 |  |  |  |  |  |  |  |
| 1 | *SCYL3* | Ordinal | rs16862745 | 168126823 | 484 | -2.22 (0.96) | 0.021 | rs1062976 | 168090284 | 0.911 | 725 | -0.183(0.102) | 0.074 |
|  |  | G/G |  |  | 237 | 19.7 |  |  |  |  | 374 | 19.2 |  |
|  |  | G/A |  |  | 211 | 18.1 |  |  |  |  | 301 | 17.7 |  |
|  |  | A/A |  |  | 36 | 14.3 |  |  |  |  | 50 | 16.2 |  |
| 1 | *MAPKAPK2* | Ordinal | rs11119389 | 204967213 | 483 | 1.90 (0.93) | 0.040 | rs4256810 | 204957072 | 1.0 | 726 | 0.064(0.096) | 0.505 |
|  |  | A/A |  |  | 240 | 16.9 |  |  |  |  | 356 | 18 |  |
|  |  | A/C |  |  | 200 | 20.9 |  |  |  |  | 296 | 18.5 |  |
|  |  | C/C |  |  | 43 | 17.7 |  |  |  |  | 74 | 19.1 |  |
| 1 | *GALNT2* | Ordinal | rs1043908 | 228483917 | 484 | 2.88 (1.28) | 0.025 | rs1043908 | 228483917 | N/A | 725 | -0.314(0.135) | 0.020 |
|  |  | A/A |  |  | 373 | 17.9 |  |  |  |  | 555 | 19 |  |
|  |  | A/G |  |  | 102 | 20.8 |  |  |  |  | 158 | 16.3 |  |
|  |  | G/G |  |  | 9 | 23.6 |  |  |  |  | 12 | 13.9 |  |
| 2 | *RHOB* | Ordinal | rs7579125 | 20512971 | 483 | 4.05 (1.87) | 0.031 |  |  |  |  |  |  |
|  |  | A/A |  |  | 430 | 18.1 |  |  |  |  |  |  |  |
|  |  | A/G |  |  | 52 | 22.5 |  |  |  |  |  |  |  |
|  |  | G/G |  |  | 1 | 17.7 |  |  |  |  |  |  |  |
| 2 | *LOC375190* | Ordinal | rs2080727 | 24204411 | 484 | 1.69 (0.85) | 0.048 | rs2080727 | 24204411 | N/A | 725 | 0.185(0.095) | 0.051 |
|  |  | A/A |  |  | 195 | 17.2 |  |  |  |  | 305 | 17.1 |  |
|  |  | A/G |  |  | 215 | 19.4 |  |  |  |  | 332 | 18.7 |  |
|  |  | G/G |  |  | 74 | 20.2 |  |  |  |  | 88 | 20.3 |  |
| 2 | *RAB10* | Ordinal | rs11677219 | 26134743 | 484 | -2.41 (0.84) | 0.004 | rs11677219 | 26134743 | N/A | 726 | -0.103(0.089) | 0.244 |
|  |  | A/A |  |  | 125 | 20.6 |  |  |  |  | 196 | 19.2 |  |
|  |  | A/G |  |  | 239 | 19 |  |  |  |  | 349 | 18.3 |  |
|  |  | G/G |  |  | 120 | 15.8 |  |  |  |  | 181 | 17.5 |  |
| 2 | *XPO1* | Ordinal | rs1050567 | 61559167 | 483 | 2.78 (1.36) | 0.042 | rs1050567 | 61559167 | N/A | 721 | -0.053(0.155) | 0.733 |
|  |  | G/G |  |  | 374 | 17.9 |  |  |  |  | 580 | 18.4 |  |
|  |  | G/A |  |  | 105 | 21 |  |  |  |  | 136 | 18 |  |
|  |  | A/A |  |  | 4 | 19.3 |  |  |  |  | 5 | 17.5 |  |
|  |  | Ordinal | rs6735330 | 61584982 | 484 | -3.59 (1.19) | 0.003 | rs6735330 | 61584982 | N/A | 726 | -0.238(0.127) | 0.061 |
|  |  | G/G |  |  | 353 | 19.7 |  |  |  |  | 530 | 19.1 |  |
|  |  | G/A |  |  | 120 | 15.9 |  |  |  |  | 182 | 17.1 |  |
|  |  | A/A |  |  | 11 | 13.3 |  |  |  |  | 14 | 15.2 |  |
| 2 | *CLASP1* | Ordinal | rs7587659 | 121940827 | 484 | -1.95 (0.97) | 0.046 |  |  |  |  |  |  |
|  |  | A/A |  |  | 251 | 19.5 |  |  |  |  |  |  |  |
|  |  | A/C |  |  | 202 | 18.3 |  |  |  |  |  |  |  |
|  |  | C/C |  |  | 31 | 14.1 |  |  |  |  |  |  |  |
|  |  | Ordinal | rs7557276 | 122035489 | 483 | 1.91 (0.85) | 0.025 | rs7557276 | 122035489 | N/A | 725 | -0.003(0.09) | 0.975 |
|  |  | A/A |  |  | 148 | 16.3 |  |  |  |  | 200 | 18.5 |  |
|  |  | A/G |  |  | 237 | 19.6 |  |  |  |  | 356 | 18.5 |  |
|  |  | G/G |  |  | 98 | 19.8 |  |  |  |  | 169 | 18.5 |  |
| 2 | *DOCK10* | Ordinal | rs1045653 | 225338679 | 484 | 2.72 (1.13) | 0.017 | rs1045653 | 225338679 | N/A | 725 | -0.08(0.117) | 0.494 |
|  |  | G/G |  |  | 316 | 17.5 |  |  |  |  | 488 | 18.5 |  |
|  |  | G/A |  |  | 157 | 20.7 |  |  |  |  | 213 | 17.8 |  |
|  |  | A/A |  |  | 11 | 20.7 |  |  |  |  | 24 | 17.1 |  |
| 3 | *RHOA* | Ordinal | rs11716445 | 49381099 | 484 | -3.48 (1.47) | 0.019 | rs11716445 | 49381099 | N/A | 726 | -0.046(0.153) | 0.761 |
|  |  | G/G |  |  | 392 | 19.3 |  |  |  |  | 597 | 18.5 |  |
|  |  | G/A |  |  | 90 | 15.8 |  |  |  |  | 123 | 18.1 |  |
|  |  | A/A |  |  | 2 | 12.2 |  |  |  |  | 6 | 17.7 |  |
| 3 | *RASSF1* | Ordinal | rs2236947 | 50346436 | 484 | -1.70 (0.86) | 0.048 | rs2236947 | 50346436 | N/A | 726 | -0.049(0.088) | 0.573 |
|  |  | C/C |  |  | 127 | 21 |  |  |  |  | 206 | 18.8 |  |
|  |  | C/A |  |  | 240 | 17.8 |  |  |  |  | 348 | 18.3 |  |
|  |  | A/A |  |  | 117 | 17.7 |  |  |  |  | 172 | 17.9 |  |
| 3 | *MCM2* | Ordinal | rs7624396 | 128813601 | 483 | -2.53 (0.96) | 0.009 | rs4974445 | 128775478 | 0.965 | 726 | 0.067(0.102) | 0.514 |
|  |  | G/G |  |  | 270 | 19.9 |  |  |  |  | 409 | 18.1 |  |
|  |  | G/A |  |  | 179 | 17.4 |  |  |  |  | 272 | 18.6 |  |
|  |  | A/A |  |  | 34 | 14.8 |  |  |  |  | 45 | 19.2 |  |
| 3 | *NEK11* | Ordinal | rs2037178 | 132384566 | 484 | 2.43 (1.10) | 0.028 | rs4682647 | 132276227 | 0.938 | 726 | 0.188(0.116) | 0.107 |
|  |  | C/C |  |  | 332 | 18.2 |  |  |  |  | 483 | 17.6 |  |
|  |  | C/A |  |  | 135 | 18.2 |  |  |  |  | 216 | 19.3 |  |
|  |  | A/A |  |  | 17 | 30.5 |  |  |  |  | 27 | 20.9 |  |
| 3 | *PAK2* | Ordinal | rs1798619 | 197970248 | 484 | -2.02 (0.97) | 0.038 | rs1798619 | 197970248 | N/A | 710 | -0.13(0.105) | 0.214 |
|  |  | A/A |  |  | 284 | 19.7 |  |  |  |  | 425 | 18.9 |  |
|  |  | A/C |  |  | 168 | 17.3 |  |  |  |  | 242 | 17.8 |  |
|  |  | C/C |  |  | 32 | 16.2 |  |  |  |  | 43 | 16.7 |  |
|  |  | Ordinal | rs4916546 | 197994930 | 484 | -2.66 (1.20) | 0.028 |  |  |  |  |  |  |
|  |  | A/A |  |  | 365 | 19.3 |  |  |  |  |  |  |  |
|  |  | A/G |  |  | 107 | 16.8 |  |  |  |  |  |  |  |
|  |  | G/G |  |  | 12 | 13.6 |  |  |  |  |  |  |  |
|  |  | Ordinal | rs2084385 | 198038264 | 483 | -2.79 (1.36) | 0.041 | rs2084385 | 198038264 | N/A | 723 | -0.048(0.14) | 0.732 |
|  |  | G/G |  |  | 375 | 19.2 |  |  |  |  | 538 | 18.7 |  |
|  |  | G/A |  |  | 104 | 17 |  |  |  |  | 178 | 18.3 |  |
|  |  | A/A |  |  | 4 | 6.3 |  |  |  |  | 7 | 17.9 |  |
| 4 | *RASSF6* | Ordinal | rs1268486 | 74676991 | 484 | 8.66 (2.92) | 0.003 | rs1247683 | 74746835 | 1.0 |  | 0.105(0.251) | 0.675 |
|  |  | G/G |  |  | 462 | 18.2 |  |  |  |  | 678 | 18.3 |  |
|  |  | G/A |  |  | 22 | 26.9 |  |  |  |  | 48 | 19.3 |  |
|  |  | A/A |  |  | 0 |  |  |  |  |  |  | 20.2 |  |
| 4 | *SEPT11* | Ordinal | rs4859727 | 78085672 | 484 | -2.39 (1.11) | 0.032 |  |  |  |  |  |  |
|  |  | A/A |  |  | 319 | 19.4 |  |  |  |  |  |  |  |
|  |  | A/G |  |  | 151 | 17.4 |  |  |  |  |  |  |  |
|  |  | G/G |  |  | 14 | 13.2 |  |  |  |  |  |  |  |
| 4 | *LSM6* | Ordinal | rs11100918 | 147331945 | 484 | -2.86 (1.26) | 0.023 | rs4835289 | 147328314 | 0.748 | 726 | -0.046(0.164) | 0.781 |
|  |  | A/A |  |  | 369 | 19.5 |  |  |  |  | 598 | 18.4 |  |
|  |  | A/G |  |  | 106 | 15.6 |  |  |  |  | 127 | 18 |  |
|  |  | G/G |  |  | 9 | 19.2 |  |  |  |  | 1 | 17.6 |  |
| 5 | *SKP2* | Ordinal | rs7731023 | 36217384 | 484 | -2.10 (0.86) | 0.015 | rs7731023 | 36217384 | N/A | 726 | -0.013(0.095) | 0.890 |
|  |  | G/G |  |  | 167 | 20.1 |  |  |  |  | 254 | 18.4 |  |
|  |  | G/A |  |  | 233 | 18.7 |  |  |  |  | 357 | 18.3 |  |
|  |  | A/A |  |  | 84 | 15.6 |  |  |  |  | 115 | 18.2 |  |
| 5 | *CETN3* | Ordinal | rs9293541 | 89722860 | 484 | -1.78 (0.92) | 0.052 |  |  |  |  |  |  |
|  |  | A/A |  |  | 248 | 19.4 |  |  |  |  |  |  |  |
|  |  | A/C |  |  | 192 | 18.7 |  |  |  |  |  |  |  |
|  |  | C/C |  |  | 44 | 14.4 |  |  |  |  |  |  |  |
| 6 | *MAPK14* | Ordinal | rs707994 | 36129239 | 484 | 2.64 (1.16) | 0.024 | rs707994 | 36129239 | N/A | 720 | -0.04(0.131) | 0.759 |
|  |  | G/G |  |  | 338 | 18 |  |  |  |  | 510 | 18.4 |  |
|  |  | G/A |  |  | 134 | 19.2 |  |  |  |  | 200 | 18 |  |
|  |  | A/A |  |  | 12 | 30.2 |  |  |  |  | 10 | 17.7 |  |
| 6 | *MCM3* | Ordinal | rs3765447 | 52249471 | 483 | 3.62 (1.76) | 0.040 | rs3765447 | 52249471 | N/A | 725 | 0.45(0.188) | 0.017 |
|  |  | A/A |  |  | 422 | 18.2 |  |  |  |  | 644 | 17.7 |  |
|  |  | A/G |  |  | 60 | 22.3 |  |  |  |  | 76 | 21.7 |  |
|  |  | G/G |  |  | 1 | 12.7 |  |  |  |  | 5 | 26.1 |  |
| 7 | *YWHAG* | Ordinal | rs11763069 | 75817615 | 483 | 6.29 (2.47) | 0.011 | rs11763069 | 75817615 | N/A | 726 | -0.37(0.224) | 0.099 |
|  |  | G/G |  |  | 452 | 18.3 |  |  |  |  | 660 | 18.6 |  |
|  |  | G/A |  |  | 31 | 24.6 |  |  |  |  | 65 | 15.5 |  |
|  |  | A/A |  |  | 0 |  |  |  |  |  | 1 | 12.7 |  |
| 7 | *MCM7* | Ordinal | rs1534309 | 99534306 | 484 | -2.02 (1.03) | 0.051 | rs941290 | 99516558 | 1.0 | 726 | -0.03(0.105) | 0.774 |
|  |  | G/G |  |  | 294 | 19.8 |  |  |  |  | 431 | 18.5 |  |
|  |  | G/C |  |  | 168 | 16.5 |  |  |  |  | 256 | 18.2 |  |
|  |  | C/C |  |  | 22 | 19.2 |  |  |  |  | 39 | 18 |  |
| 8 | *TNKS* | Ordinal | rs9329203 | 9449563 | 484 | -2.09 (0.99) | 0.036 | rs17150237 | 9449129 | 0.898 | 725 | 0.101(0.107) | 0.345 |
|  |  | A/A |  |  | 247 | 19.7 |  |  |  |  | 410 | 18.2 |  |
|  |  | A/G |  |  | 208 | 17.8 |  |  |  |  | 279 | 19.1 |  |
|  |  | G/G |  |  | 29 | 15.4 |  |  |  |  | 36 | 20 |  |
|  |  | Ordinal | rs12549064 | 9479437 | 484 | -2.15 (1.09) | 0.048 | rs12549064 | 9479437 | N/A | 725 | 0.321(0.116) | 0.006 |
|  |  | A/A |  |  | 303 | 19.5 |  |  |  |  | 496 | 17.8 |  |
|  |  | A/C |  |  | 166 | 17.4 |  |  |  |  | 205 | 20.6 |  |
|  |  | C/C |  |  | 15 | 15.1 |  |  |  |  | 24 | 23.6 |  |
|  |  | Ordinal | rs12679892 | 9482839 | 484 | -1.80 (0.92) | 0.051 | rs12679892 | 9482839 | N/A | 726 | 0.184(0.1) | 0.066 |
|  |  | A/A |  |  | 218 | 20.1 |  |  |  |  | 339 | 17.6 |  |
|  |  | A/G |  |  | 216 | 17.4 |  |  |  |  | 326 | 19.2 |  |
|  |  | G/G |  |  | 50 | 17.6 |  |  |  |  | 61 | 20.8 |  |
| 8 | *DOCK5* | Ordinal | rs2659582 | 25325910 | 484 | 2.03 (0.87) | 0.021 | rs11135869 | 25391271 | 0.934 | 725 | 0.023(0.098) | 0.814 |
|  |  | A/A |  |  | 188 | 16.7 |  |  |  |  | 304 | 18.2 |  |
|  |  | A/G |  |  | 225 | 19.8 |  |  |  |  | 343 | 18.4 |  |
|  |  | G/G |  |  | 71 | 20.1 |  |  |  |  | 78 | 18.6 |  |
|  |  | Ordinal | rs7008627 | 25326425 | 484 | 2.01 (0.87) | 0.022 | rs11135869 | 25391271 | 0.934 | 725 | 0.023(0.098) | 0.814 |
|  |  | G/G |  |  | 190 | 16.7 |  |  |  |  | 304 | 18.2 |  |
|  |  | G/A |  |  | 223 | 19.8 |  |  |  |  | 343 | 18.4 |  |
|  |  | A/A |  |  | 71 | 20.1 |  |  |  |  | 78 | 18.6 |  |
|  |  | Ordinal | rs301486 | 4670653 | 484 | -3.25 (1.18) | 0.006 | rs301486 | 4670653 | N/A | 726 | -0.105(0.13) | 0.417 |
|  |  | G/G |  |  | 338 | 19.6 |  |  |  |  | 539 | 18.5 |  |
|  |  | G/A |  |  | 136 | 16.9 |  |  |  |  | 173 | 17.6 |  |
|  |  | A/A |  |  | 10 | 10.2 |  |  |  |  | 14 | 16.7 |  |
| 9 | *CDC37L1* | Ordinal | rs367147 | 4683092 | 484 | 1.89 (0.86) | 0.029 | rs1331191 | 4694420 | 1.0 | 726 | 0.098(0.09) | 0.275 |
|  |  | G/G |  |  | 132 | 16.9 |  |  |  |  | 206 | 17.5 |  |
|  |  | G/C |  |  | 246 | 18.6 |  |  |  |  | 355 | 18.3 |  |
|  |  | C/C |  |  | 106 | 20.7 |  |  |  |  | 165 | 19.2 |  |
| 9 | *AK3* | Ordinal | rs6915 | 4701440 | 484 | -1.86 (0.83) | 0.026 |  |  |  |  |  |  |
|  |  | A/A |  |  | 116 | 20.6 |  |  |  |  |  |  |  |
|  |  | A/T |  |  | 232 | 18.7 |  |  |  |  |  |  |  |
|  |  | T/T |  |  | 136 | 16.8 |  |  |  |  |  |  |  |
| 9 | *PRPF4* | Ordinal | rs10116039 | 115078001 | 482 | -2.29 (0.95) | 0.016 | rs10116039 | 115078001 | N/A | 726 | -0.098(0.101) | 0.331 |
|  |  | G/G |  |  | 246 | 19.7 |  |  |  |  | 391 | 18.9 |  |
|  |  | G/A |  |  | 195 | 18.5 |  |  |  |  | 282 | 18 |  |
|  |  | A/A |  |  | 41 | 13.4 |  |  |  |  | 53 | 17.2 |  |
|  |  | Ordinal | **rs10733604** | **115082084** | **484** | **5.84 (1.12)** | **<0.0001** | **rs10733604** | **115082084** | **N/A** | **726** | **0.14(0.115)** | **0.227** |
|  |  | C/C |  |  | **344** | **17** |  |  |  |  | **472** | **17.9** |  |
|  |  | C/A |  |  | **126** | **21.5** |  |  |  |  | **230** | **19.1** |  |
|  |  | A/A |  |  | **14** | **33.7** |  |  |  |  | **24** | **20.4** |  |
| 9 | *CEP110* | Ordinal | rs10818504 | 122900510 | 484 | 1.74 (0.86) | 0.042 | rs10818504 | 122900510 | N/A | 726 | 0.025(0.092) | 0.784 |
|  |  | G/G |  |  | 147 | 17.3 |  |  |  |  | 249 | 18.2 |  |
|  |  | G/A |  |  | 239 | 18.5 |  |  |  |  | 361 | 18.4 |  |
|  |  | A/A |  |  | 98 | 20.9 |  |  |  |  | 116 | 18.6 |  |
| 9 | *NEK6* | Ordinal | rs4838143 | 126057457 | 484 | 2.69 (1.15) | 0.020 | rs4838140 | 126049935 | 1.0 | 724 | 0.012(0.119) | 0.920 |
|  |  | C/C |  |  | 339 | 17.7 |  |  |  |  | 476 | 18.3 |  |
|  |  | C/G |  |  | 132 | 20.5 |  |  |  |  | 226 | 18.4 |  |
|  |  | G/G |  |  | 13 | 22.6 |  |  |  |  | 22 | 18.5 |  |
|  |  | Ordinal | rs4838158 | 126107997 | 484 | 3.03 (1.30) | 0.021 | rs17222478 | 126105657 | 0.867 | 725 | 0.013(0.132) | 0.923 |
|  |  | A/A |  |  | 374 | 17.9 |  |  |  |  | 517 | 18.4 |  |
|  |  | A/G |  |  | 103 | 21.1 |  |  |  |  | 197 | 18.5 |  |
|  |  | G/G |  |  | 7 | 22.9 |  |  |  |  | 11 | 18.6 |  |
|  |  | Ordinal | rs4838159 | 126109450 | 484 | 2.25 (1.11) | 0.042 | rs4838159 | 126109450 | N/A | 726 | -0.069(0.113) | 0.543 |
|  |  | A/A |  |  | 312 | 17.8 |  |  |  |  | 445 | 18.6 |  |
|  |  | A/C |  |  | 158 | 19.7 |  |  |  |  | 253 | 18 |  |
|  |  | C/C |  |  | 14 | 24 |  |  |  |  | 28 | 17.5 |  |
| 10 | *RASSF4* | Ordinal | rs883734 | 44788195 | 484 | 1.88 (0.90) | 0.036 | rs3740098 | 44791228 | 1.0 | 725 | -0.026(0.093) | 0.780 |
|  |  | T/T |  |  | 208 | 17.5 |  |  |  |  | 316 | 18.5 |  |
|  |  | T/A |  |  | 220 | 18.9 |  |  |  |  | 319 | 18.3 |  |
|  |  | A/A |  |  | 56 | 21.6 |  |  |  |  | 90 | 18.1 |  |
|  |  | Ordinal | rs870957 | 44798098 | 484 | 2.51 (1.14) | 0.028 | rs870957 | 44798098 | N/A | 725 | 0.021(0.124) | 0.868 |
|  |  | A/A |  |  | 353 | 17.8 |  |  |  |  | 528 | 18.3 |  |
|  |  | A/G |  |  | 115 | 20.7 |  |  |  |  | 178 | 18.5 |  |
|  |  | G/G |  |  | 16 | 21.6 |  |  |  |  | 19 | 18.6 |  |
|  |  | Ordinal | rs7896801 | 44801458 | 484 | 2.12 (0.98) | 0.031 | rs7896801 | 44801458 | N/A | 726 | -0.072(0.101) | 0.480 |
|  |  | A/A |  |  | 267 | 17.8 |  |  |  |  | 400 | 18.7 |  |
|  |  | A/G |  |  | 187 | 19 |  |  |  |  | 276 | 18.1 |  |
|  |  | G/G |  |  | 30 | 23.7 |  |  |  |  | 50 | 17.5 |  |
|  |  | Ordinal | rs2297492 | 44811377 | 484 | 4.23 (1.49) | 0.005 | rs2297492 | 44811377 | N/A | 726 | 0.213(0.146) | 0.144 |
|  |  | A/A |  |  | 407 | 17.9 |  |  |  |  | 591 | 17.9 |  |
|  |  | A/G |  |  | 72 | 22.4 |  |  |  |  | 127 | 19.8 |  |
|  |  | G/G |  |  | 5 | 24.3 |  |  |  |  | 8 | 21.7 |  |
| 10 | *CDC2* | Ordinal | rs3213025 | 62209197 | 484 | 3.18 (1.14) | 0.005 |  |  |  |  |  |  |
|  |  | A/A |  |  | 347 | 17.7 |  |  |  |  |  |  |  |
|  |  | A/G |  |  | 121 | 20.1 |  |  |  |  |  |  |  |
|  |  | G/G |  |  | 16 | 26.7 |  |  |  |  |  |  |  |
|  |  | Ordinal | rs3213077 | 62222738 | 483 | 2.48 (1.06) | 0.020 | rs3213077 | 62222738 | N/A | 725 | 0.04(0.115) | 0.731 |
|  |  | A/A |  |  | 325 | 18 |  |  |  |  | 458 | 18.1 |  |
|  |  | A/G |  |  | 136 | 19 |  |  |  |  | 244 | 18.5 |  |
|  |  | G/G |  |  | 22 | 26.4 |  |  |  |  | 23 | 18.8 |  |
| 10 | *KIF11* | Ordinal | rs2275220 | 94362686 | 484 | -4.23 (2.12) | 0.046 | rs2275220 | 94362686 | N/A | 726 | 0.45(0.172) | 0.009 |
|  |  | A/A |  |  | 448 | 19 |  |  |  |  | 613 | 17.9 |  |
|  |  | A/G |  |  | 34 | 13.5 |  |  |  |  | 111 | 22 |  |
|  |  | G/G |  |  | 2 | 20.4 |  |  |  |  | 2 | 26.4 |  |
| 10 | *TACC2* | Ordinal | rs1885516 | 123910774 | 484 | 3.29 (1.35) | 0.015 | rs1885516 | 123910774 | N/A | 726 | 0.15(0.155) | 0.332 |
|  |  | A/A |  |  | 387 | 17.8 |  |  |  |  | 593 | 18.1 |  |
|  |  | A/C |  |  | 90 | 22.4 |  |  |  |  | 127 | 19.4 |  |
|  |  | C/C |  |  | 7 | 17 |  |  |  |  | 6 | 20.7 |  |
| 11 | *STIM1* | Ordinal | rs3794050 | 4068476 | 484 | 2.85 (1.38) | 0.039 | rs3794050 | 4068476 | N/A | 726 | -0.383(0.142) | 0.007 |
|  |  | G/G |  |  | 389 | 17.8 |  |  |  |  | 547 | 19.4 |  |
|  |  | G/A |  |  | 89 | 23 |  |  |  |  | 173 | 16.2 |  |
|  |  | A/A |  |  | 6 | 8.6 |  |  |  |  | 6 | 13.3 |  |
| 11 | *KIAA0999* | Ordinal | rs681524 | 116253524 | 484 | -3.33 (1.71) | 0.051 | rs681524 | 116253524 | N/A | 726 | -0.065(0.174) | 0.708 |
|  |  | A/A |  |  | 414 | 19.1 |  |  |  |  | 629 | 18.5 |  |
|  |  | A/G |  |  | 70 | 15.8 |  |  |  |  | 93 | 17.9 |  |
|  |  | G/G |  |  | 0 |  |  |  |  |  | 4 | 17.4 |  |
|  |  | Ordinal | rs17120197 | 116350314 | 484 | 2.39 (1.03) | 0.020 | rs17120197 | 116350314 | N/A | 726 | -0.159(0.114) | 0.163 |
|  |  | A/A |  |  | 304 | 17.7 |  |  |  |  | 476 | 18.9 |  |
|  |  | A/G |  |  | 157 | 19.9 |  |  |  |  | 223 | 17.6 |  |
|  |  | G/G |  |  | 23 | 22.8 |  |  |  |  | 27 | 16.3 |  |
|  |  | Ordinal | rs12269901 | 116479139 | 483 | 1.91 (0.92) | 0.038 | rs723955 | 116430252 | 0.762 | 726 | 0.071(0.109) | 0.512 |
|  |  | C/C |  |  | 233 | 17.7 |  |  |  |  | 428 | 18.1 |  |
|  |  | C/G |  |  | 205 | 18.7 |  |  |  |  | 263 | 18.7 |  |
|  |  | G/G |  |  | 45 | 22.7 |  |  |  |  | 35 | 19.3 |  |
| 11 | *JAM3* | Ordinal | rs610829 | 133523955 | 484 | 3.62 (1.74) | 0.038 |  |  |  |  |  |  |
|  |  | A/A |  |  | 425 | 18.1 |  |  |  |  |  |  |  |
|  |  | A/G |  |  | 57 | 22.7 |  |  |  |  |  |  |  |
|  |  | G/G |  |  | 2 | 11.8 |  |  |  |  |  |  |  |
| 12 | *ESPL1* | Ordinal | rs1110720 | 51968593 | 484 | -1.67 (0.86) | 0.053 | rs4759021 | 52001135 | 0.964 | 726 | -0.005(0.091) | 0.952 |
|  |  | A/A |  |  | 190 | 20.5 |  |  |  |  | 319 | 18.4 |  |
|  |  | A/G |  |  | 219 | 17.1 |  |  |  |  | 304 | 18.4 |  |
|  |  | G/G |  |  | 75 | 18.2 |  |  |  |  | 103 | 18.3 |  |
| 14 | *MNAT1* | Ordinal | rs2104075 | 60362496 | 483 | -2.70 (1.29) | 0.037 |  |  |  |  |  |  |
|  |  | A/A |  |  | 375 | 19.2 |  |  |  |  |  |  |  |
|  |  | A/T |  |  | 99 | 16.9 |  |  |  |  |  |  |  |
|  |  | T/T |  |  | 9 | 11.8 |  |  |  |  |  |  |  |
|  |  | Ordinal | rs12888332 | 60430848 | 484 | -6.15 (2.27) | 0.007 | rs12888332 | 60430848 | N/A | 726 | 0.101(0.238) | 0.671 |
|  |  | A/A |  |  | 448 | 19.1 |  |  |  |  | 675 | 18.3 |  |
|  |  | A/C |  |  | 36 | 12.9 |  |  |  |  | 49 | 19.2 |  |
|  |  | C/C |  |  | 0 |  |  |  |  |  | 2 | 20.1 |  |
|  |  | Ordinal | rs4151330 | 60441298 | 483 | -2.43 (0.95) | 0.010 | rs4151330 | 60441298 | N/A | 720 | -0.052(0.095) | 0.584 |
|  |  | A/A |  |  | 229 | 20.4 |  |  |  |  | 308 | 18.9 |  |
|  |  | A/G |  |  | 215 | 17.1 |  |  |  |  | 330 | 18.4 |  |
|  |  | G/G |  |  | 39 | 17 |  |  |  |  | 82 | 18 |  |
| 14 | *FAM164C* | Ordinal | rs12431669 | 74615983 | 484 | 2.08 (0.93) | 0.026 | rs12431669 | 74615983 | N/A | 726 | 0.09(0.101) | 0.374 |
|  |  | G/G |  |  | 249 | 17.2 |  |  |  |  | 373 | 17.9 |  |
|  |  | G/A |  |  | 194 | 20.3 |  |  |  |  | 297 | 18.6 |  |
|  |  | A/A |  |  | 41 | 19.8 |  |  |  |  | 56 | 19.4 |  |
| 15 | *PAK6* | Ordinal | rs900055 | 38319876 | 484 | 1.78 (0.83) | 0.031 | rs1017843 | 38315447 | 0.935 | 726 | -0.066(0.09) | 0.463 |
|  |  | A/A |  |  | 134 | 16.9 |  |  |  |  | 219 | 18.8 |  |
|  |  | A/G |  |  | 230 | 18.7 |  |  |  |  | 362 | 18.3 |  |
|  |  | G/G |  |  | 120 | 20.4 |  |  |  |  | 145 | 17.7 |  |
|  |  | Ordinal | rs2068001 | 38337441 | 483 | 1.90 (0.88) | 0.031 | rs2068001 | 38337441 | N/A | 726 | -0.043(0.094) | 0.644 |
|  |  | G/G |  |  | 197 | 17.3 |  |  |  |  | 291 | 18.6 |  |
|  |  | G/A |  |  | 220 | 19 |  |  |  |  | 340 | 18.2 |  |
|  |  | A/A |  |  | 66 | 21.3 |  |  |  |  | 95 | 17.9 |  |
|  |  | Ordinal | rs2242119 | 38356115 | 484 | 2.18 (0.83) | 0.009 | rs2242119 | 38356115 | N/A | 726 | 0.081(0.091) | 0.377 |
|  |  | C/C |  |  | 199 | 17.1 |  |  |  |  | 275 | 17.9 |  |
|  |  | C/A |  |  | 204 | 18.9 |  |  |  |  | 339 | 18.5 |  |
|  |  | A/A |  |  | 81 | 21.7 |  |  |  |  | 112 | 19.2 |  |
| 15 | *LOC100131244* | Ordinal | rs4924445 | 38360705 | 484 | 1.75 (0.86) | 0.042 | rs4924445 | 38360705 | N/A | 724 | 0.038(0.092) | 0.684 |
|  |  | G/G |  |  | 200 | 17 |  |  |  |  | 274 | 18.1 |  |
|  |  | G/A |  |  | 212 | 19.7 |  |  |  |  | 340 | 18.4 |  |
|  |  | A/A |  |  | 72 | 19.9 |  |  |  |  | 110 | 18.7 |  |
| 17 | *CNTROB* | Ordinal | rs4462665 | 7789812 | 484 | -1.81 (0.85) | 0.033 | rs11650083 | 7788680 | 1.0 | 726 | -0.111(0.09) | 0.220 |
|  |  | G/G |  |  | 140 | 20.5 |  |  |  |  | 192 | 19.1 |  |
|  |  | G/C |  |  | 237 | 18.2 |  |  |  |  | 359 | 18.2 |  |
|  |  | C/C |  |  | 107 | 17 |  |  |  |  | 175 | 17.2 |  |
|  |  | Ordinal | rs7217194 | 7793612 | 483 | -1.71 (0.85) | 0.045 | rs11650083 | 7788680 | 0.966 | 726 | -0.111(0.09) | 0.220 |
|  |  | A/A |  |  | 126 | 20.2 |  |  |  |  | 192 | 19.1 |  |
|  |  | A/C |  |  | 241 | 18.8 |  |  |  |  | 359 | 18.2 |  |
|  |  | C/C |  |  | 116 | 16.8 |  |  |  |  | 175 | 17.2 |  |
| 17 | *AURKB* | Ordinal | rs3027260 | 8053911 | 484 | -3.33 (1.52) | 0.029 | rs3027260 | 8053911 | N/A | 726 | -0.404(0.177) | 0.022 |
|  |  | G/G |  |  | 404 | 19.2 |  |  |  |  | 632 | 18.7 |  |
|  |  | G/A |  |  | 77 | 15.8 |  |  |  |  | 91 | 15.4 |  |
|  |  | A/A |  |  | 3 | 12.8 |  |  |  |  | 3 | 12.4 |  |
|  |  | Ordinal | rs4792590 | 8057840 | 484 | -3.14 (1.34) | 0.020 | rs4792590 | 8057840 | N/A | 726 | -0.39(0.152) | 0.010 |
|  |  | G/G |  |  | 377 | 19.3 |  |  |  |  | 597 | 18.6 |  |
|  |  | G/A |  |  | 102 | 16.6 |  |  |  |  | 121 | 15.4 |  |
|  |  | A/A |  |  | 5 | 9.5 |  |  |  |  | 8 | 12.5 |  |
| 17 | *NLK* | Ordinal | rs17792426 | 23487016 | 484 | 2.34 (0.94) | 0.014 | rs17792426 | 23487016 | N/A | 726 | -0.101(0.1) | 0.314 |
|  |  | A/A |  |  | 229 | 17 |  |  |  |  | 383 | 18.8 |  |
|  |  | A/G |  |  | 215 | 19.9 |  |  |  |  | 283 | 18 |  |
|  |  | G/G |  |  | 40 | 20.9 |  |  |  |  | 60 | 17.1 |  |
| 17 | *YPEL2* | Ordinal | rs10853011 | 54803764 | 484 | 2.68 (1.23) | 0.031 | rs2290266 | 54821699 | 1.0 | 726 | 0.038(0.129) | 0.771 |
|  |  | G/G |  |  | 360 | 18 |  |  |  |  | 529 | 18.3 |  |
|  |  | G/A |  |  | 115 | 20.2 |  |  |  |  | 182 | 18.6 |  |
|  |  | A/A |  |  | 9 | 25.6 |  |  |  |  | 15 | 18.9 |  |
| 18 | *CABLES1* | Ordinal | rs6507528 | 19025578 | 484 | -2.54 (0.83) | 0.002 |  |  |  |  |  |  |
|  |  | A/A |  |  | 151 | 21 |  |  |  |  |  |  |  |
|  |  | A/G |  |  | 227 | 18.3 |  |  |  |  |  |  |  |
|  |  | G/G |  |  | 106 | 15.9 |  |  |  |  |  |  |  |
|  |  | Ordinal | rs1968470 | 19082970 | 484 | 2.46 (0.90) | 0.007 | rs1968470 | 19082970 | N/A | 726 | 0.047(0.093) | 0.616 |
|  |  | A/A |  |  | 184 | 16.9 |  |  |  |  | 301 | 18.1 |  |
|  |  | A/G |  |  | 238 | 19 |  |  |  |  | 329 | 18.5 |  |
|  |  | G/G |  |  | 62 | 22.1 |  |  |  |  | 96 | 18.9 |  |
|  |  | Ordinal | rs2164029 | 19085007 | 484 | 1.84 (0.88) | 0.038 | rs2164029 | 19085007 | N/A | 726 | 0.018(0.094) | 0.850 |
|  |  | A/A |  |  | 193 | 17.3 |  |  |  |  | 297 | 18.2 |  |
|  |  | A/G |  |  | 226 | 19.1 |  |  |  |  | 337 | 18.4 |  |
|  |  | G/G |  |  | 65 | 20.9 |  |  |  |  | 92 | 18.6 |  |
| 20 | *SNRPB* | Ordinal | rs6049212 | 2390022 | 484 | -3.28 (1.34) | 0.015 | rs6049212 | 2390022 | N/A | 726 | 0.104(0.14) | 0.460 |
|  |  | G/G |  |  | 381 | 19.4 |  |  |  |  | 563 | 18.1 |  |
|  |  | G/A |  |  | 97 | 15.8 |  |  |  |  | 154 | 19 |  |
|  |  | A/A |  |  | 6 | 14.7 |  |  |  |  | 9 | 19.9 |  |
| 20 | *RASSF2* | Ordinal | rs723561 | 4725740 | 484 | -3.93 (1.64) | 0.017 | rs723561 | 4725740 | N/A | 726 | 0.004(0.192) | 0.982 |
|  |  | G/G |  |  | 419 | 19.2 |  |  |  |  | 640 | 18.4 |  |
|  |  | G/A |  |  | 62 | 14.7 |  |  |  |  | 84 | 18.4 |  |
|  |  | A/A |  |  | 3 | 17 |  |  |  |  | 2 | 18.4 |  |
| 20 | *STK4* | Ordinal | rs2868218 | 43025662 | 483 | 2.34 (1.09) | 0.033 | rs2868218 | 43025662 | N/A | 726 | -0.113(0.11) | 0.307 |
|  |  | A/A |  |  | 316 | 17.7 |  |  |  |  | 443 | 18.9 |  |
|  |  | A/G |  |  | 151 | 20.1 |  |  |  |  | 250 | 18 |  |
|  |  | G/G |  |  | 16 | 22.1 |  |  |  |  | 33 | 17 |  |
| 20 | *CABLES2* | Ordinal | rs6121396 | 60398024 | 484 | -4.09 (1.61) | 0.011 | rs13043447 | 60398252 | 1.0 | 726 | 0.311(0.188) | 0.099 |
|  |  | A/A |  |  | 412 | 19.3 |  |  |  |  | 642 | 18 |  |
|  |  | A/G |  |  | 70 | 14.6 |  |  |  |  | 82 | 20.8 |  |
|  |  | G/G |  |  | 2 | 20.9 |  |  |  |  | 2 | 23.7 |  |
| 22 | *WBP2NL* | Ordinal | rs133306 | 40726096 | 483 | -1.86 (0.84) | 0.028 | rs133335 | 40746002 | 1.0 | 726 | 0.115(0.088) | 0.190 |
|  |  | G/G |  |  | 136 | 19.9 |  |  |  |  | 197 | 17.3 |  |
|  |  | G/A |  |  | 238 | 18.8 |  |  |  |  | 344 | 18.3 |  |
|  |  | A/A |  |  | 109 | 16.1 |  |  |  |  | 185 | 19.3 |  |
| 23 | *SEPT6* | Ordinal | rs11797680 | 118659972 | 483 | -2.44 (1.05) | 0.021 |  |  |  |  |  |  |
|  |  | A/A |  |  | 291 | 19.7 |  |  |  |  |  |  |  |
|  |  | A/G |  |  | 173 | 17.1 |  |  |  |  |  |  |  |
|  |  | G/G |  |  | 19 | 15.5 |  |  |  |  |  |  |  |
|  |  | Ordinal | rs2107899 | 118669079 | 484 | -2.71 (1.11) | 0.015 |  |  |  |  |  |  |
|  |  | A/A |  |  | 319 | 19.5 |  |  |  |  |  |  |  |
|  |  | A/C |  |  | 151 | 17.3 |  |  |  |  |  |  |  |
|  |  | C/C |  |  | 14 | 12.2 |  |  |  |  |  |  |  |
| 23 | *MPP1* | Ordinal | rs2728729 | 153662541 | 483 | 2.00 (0.99) | 0.044 |  |  |  |  |  |  |
|  |  | A/A |  |  | 256 | 17.2 |  |  |  |  |  |  |  |
|  |  | A/G |  |  | 199 | 20.6 |  |  |  |  |  |  |  |
|  |  | G/G |  |  | 28 | 17.9 |  |  |  |  |  |  |  |
| ^a^If blank, genotype information was not available in SASBAC (n=15 SNPs).  ^b^PMD Est(SE): ordinal parameter estimate and standard error reflecting the estimated change in percent density per each additional copy of the minor allele carried . Adjusted mean from general model: least squares estimate of mean percent density for each genotype, adjusted for age, BMI, and PMH. ^c^p-value from analyses adjusted for age, BMI, and PMH. ^d^Sqrt PMD Est(SE): ordinal parameter estimate and standard error reflecting the estimated change in percent density error per each additional copy of the minor allele carried. Adjusted mean from general model: least squares estimate of mean percent density for each genotype, adjusted for age, BMI, and PMH. Least squares means back transformed from square root to original scale. | | | | | | | | | | | | | |
